# Supplementary material for: Autophagy-enhancing ATG16L1 polymorphism is associated with improved clinical outcome and T-cell immunity in chronic HIV-1 infection
Source: Nat Commun. 2024 Mar 28;15:2465. doi: 10.1038/s41467-024-46606-z (PMC10979031; doi:10.1038/s41467-024-46606-z)
Supplement: Supplementary file 1 — Supplementary Information [file 41467_2024_46606_MOESM1_ESM.pdf]

SNP name	AIDS *p*	AIDS RH	95% CI for AIDS RH	Death *p*	Death RH	95% CI for Death RH
rs6861	0.021	2.455	1.143-5.277	0.012	2.874	1.262-6.543
CCR5Δ32	0.0001	2.461	1.556-3.893	0.0002	2.476	3.979-1.541
rs6861	0.010	2.724	1.274-5.828	0.006	3.173	1.400-7.193
HLA-B57	0.006	4.053	1.500-10.972	0.013	3.540	1.308-9.576

Supplementary Table 1. The effect of *ATG16L1* rs6861 is independent of the CCR5Δ32 and HLA-B57 genotypes. *p*-values indicate multivariate survival analyses, comparing rs6861 to either CCR5Δ32 or HLA-B57, using Cox regression with endpoints AIDS (CDC1987) or AIDS-related death in HIV-1-infected individuals. RH = relative hazard, CI = confidence interval. See Fig. 1.

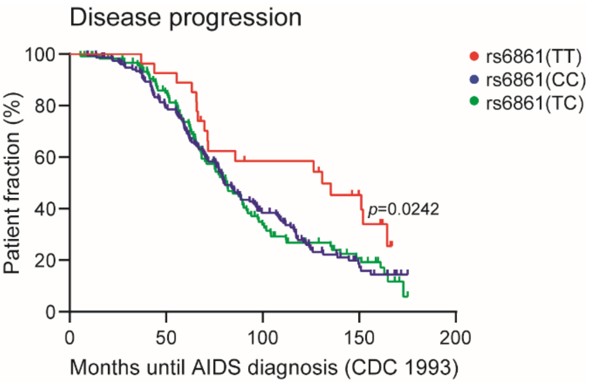

Supplementary Fig. 1. *ATG16L1* rs6861(TT) genetic variant is associated with delayed disease progression in HIV-1-infected individuals. Survival analysis using the endpoint AIDS diagnosis (CDC 1993) comparing rs6861(TT) HIV-1 infected individuals (n=27) to combined rs6861(CC) (n=122) and rs6861(CT) (n=155) variants. Kaplan Meier survival analysis (Log-Rank test). See Fig. 2. Source data are provided as a Supplementary Source Data file.

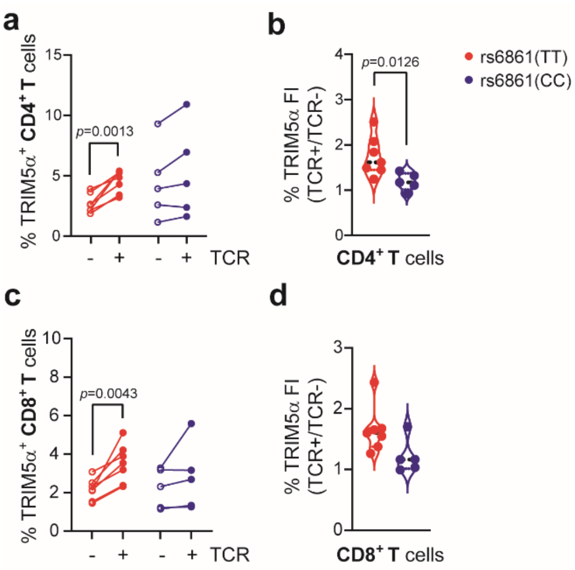

Supplementary Fig. 2. Frequency of TRIM5α+ CD4+ and CD8+ T cells upon TCR stimulation. a,c Percentage TRIM5α+ CD4+ T cells (a) or TRIM5α+ CD8+ T cells (c) at steady state (-) or upon TCR-engagement using soluble anti-CD3 and anti-CD28 (+) (a,c dependent two-tailed *t*-test). **b,d** Fold increase (FI) of percentage TRIM5α+ CD4+ T cells (b) or TRIM5α+ CD8+ T cells (d) upon TCR-engagement (b,d independent two-tailed *t*-test) (a-d TT n=7, CC n=5). See Fig. 3. Source data are provided as a Supplementary Source Data file.

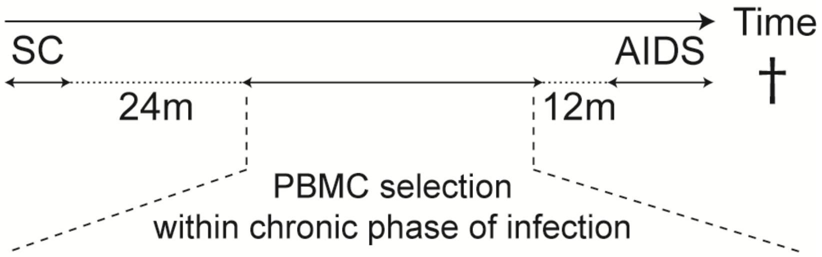

Donor ID	rs6861	Sex	Type	SC year	SC-to-PBMC (y)	PBMC-to-AIDS (y)	CD4 count/mL
1	TT	M	MSM	1988	5	1.4	>400
2	TT	M	MSM	1983	6	5.2	>400
3	TT	M	MSM	1983	9	3.6[#]	>400
4	TT	M	MSM	1986	3	4.3	>400
5	TT	M	MSM	1990	4	1.3	>400
6	TT	M	MSM	1986	4	6.7[#]	>400
7	CC	M	MSM	1983	5	1.3	>400
8	CC	M	MSM	1983	9	4.2[#]	>400
9	CC	M	MSM	1986	2	7.6	>400
10	CC	M	MSM	1983	6	1.5	>400
11	CC	M	MSM	1983	7	3.1[#]	>400
12	CC	M	MSM	1989	4	3.1[#]	>400

Supplementary Fig. 3. Clinical characteristics and PBMCs selection of HIV-1-infected individuals from the ACS cohort during the chronic phase of infection. Twelve HIV-1-infected MSM at chronic stages of infection (Donor ID 1-6, genotype rs6861(TT); 7-12, genotyped rs6861(CC)) were selected between >24 months after seroconversion (SC) and >12 months prior to AIDS diagnosis. Sex, year of seroconversion, CD4 count and time between seroconversion, sample selection, and aids diagnosis are depicted in this table and indicate high similarity between the six selected PBMC samples of the two genotypes. Age at seroconversion of the donors was between 25-38 years old. ACS = Amsterdam Cohort Studies, SC = seroconversion, y = years, # = AIDS-free follow-up after SC.

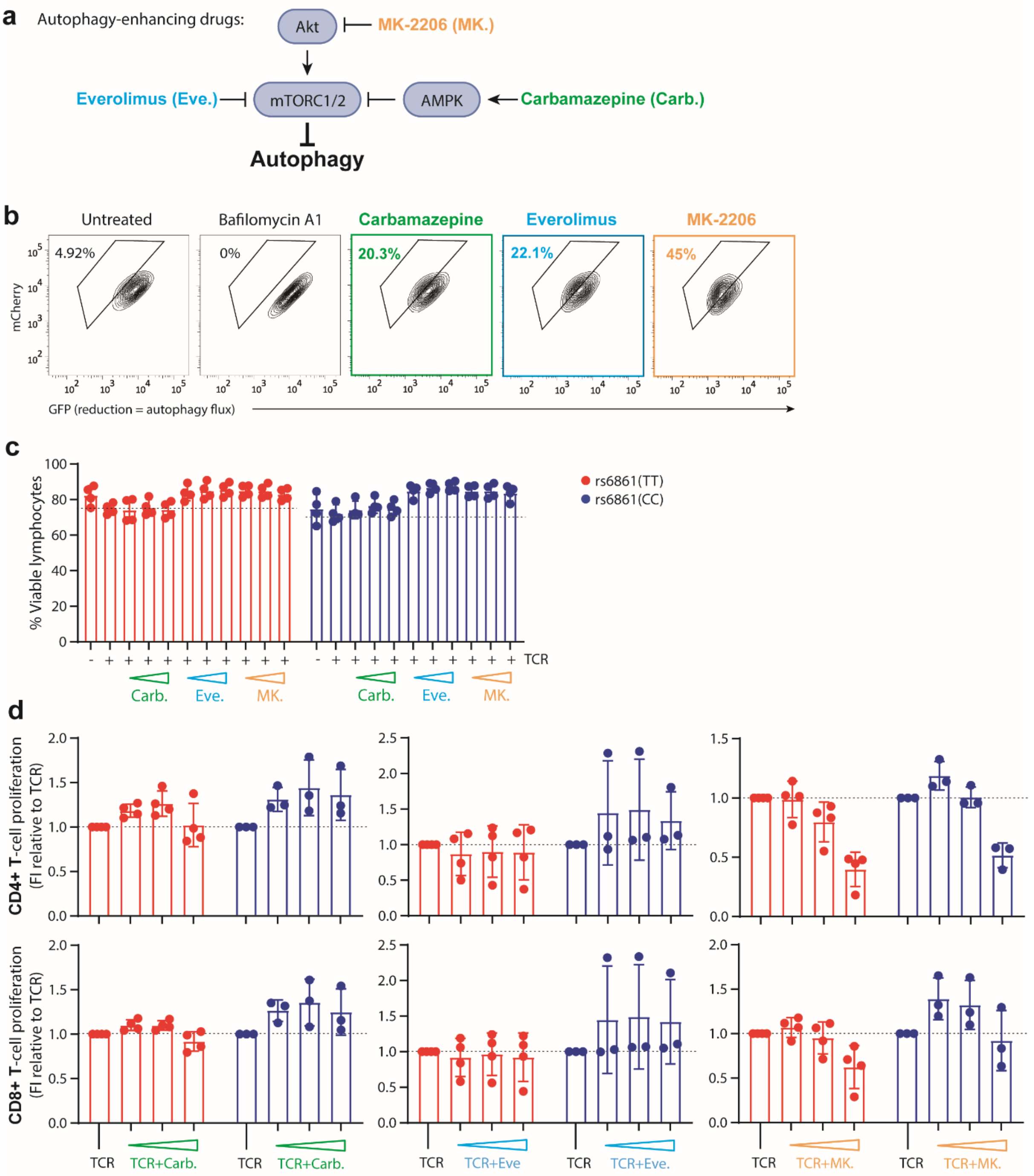

Supplementary Fig. 4. Selection and dosage of autophagy-enhancing therapeutics. a Schematic representation of the three autophagy-enhancing drugs and their molecular targets utilized to pharmaceutically increase autophagy flux in this manuscript. **b** Verification of enhanced autophagic flux in response to treatment with carbamazepine (Carb. [100 µM]), everolimus (Eve. [3 nM]), and MK-2206 (MK. [5 uM]) in U87 mcherry-GFP-LC3 reporter cells. The ratio of mCherry to GFP indicates the level of autophagy flux. **c** A concentration range of the three autophagy-enhancing drugs (Carb., 50, 100, 200 µM; Eve., 2.5, 5, 10 nM; MK., 0.5, 1, 2 µM) demonstrates no impact on lymphocyte viability in combination with TCR-engagement as determined by flow cytometry (TT n=4, CC n=4). **d** Impact of drug concentrations (Carb., 50, 100, 200 µM; Eve., 2.5, 5, 10 nM; MK., 0.5, 1, 2 µM) on CD4+ and CD8+ T-cell proliferation indicates opposing effect of drugs in rs6861(TT) (n=4) versus rs6861(CC) (n=3) genotyped individuals at various concentrations. Concentrations selected for further studies were 100 uM for Carb., 5 nM for Eve., and 1 uM for MK. Dashed lines (c,d) represents T-cell response to TCR-stimulation without autophagy drugs. Graphs (c,d) represent mean ± SD. CTV = CellTrace Violet, TCR = anti-CD3/CD28 stimulation, Carb. = carbamazepine, Eve. = everolimus, MK. = MK-2206. Source data are provided as a Supplementary Source Data file.

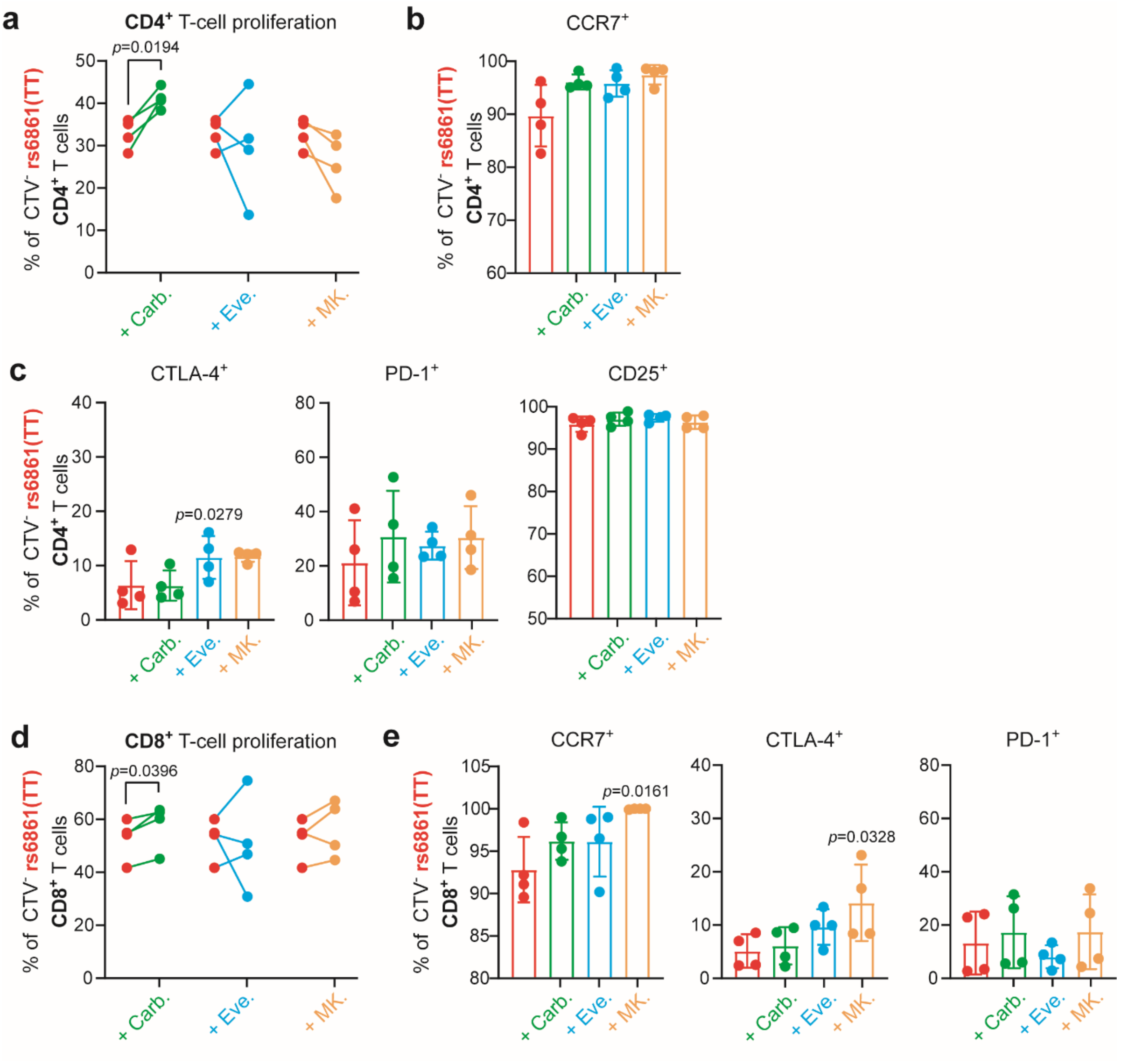

Supplementary Fig. 5. Impact of autophagy-enhancing drugs on rs6861(TT) CD4+ and CD8+ T-cell phenotype and function. PBMCs derived from healthy rs6861(TT) individuals were treated with anti-CD3/CD28 in combination with autophagy-enhancing drugs carbamezapine (Carb., 100 µM), everolimus (Eve., 5 nM) and MK-2206 (MK. 1 µM) for 4 days, after which the effect of the drug treatement was evaluated on **a** CD4+ T-cell proliferation, **b** frequency of CCR7+ CD4+ T cells, **c** frequency of CTLA-4+, PD-1+ and CD25+ CD4+ T cells, **d** CD8+ T-cell proliferation, **e** frequency of CCR7+, CTLA-4+ and PD-1+ CD8+ T cells (a-e n=4). Graphs (b-e) represent mean ± SD. CTV = CellTrace Violet, Carb. = carbamazepine, Eve. = everolimus, MK. = MK-2206. Statistical significances were determined using dependent two-tailed *t*-tests. Source data are provided as a Supplementary Source Data file.

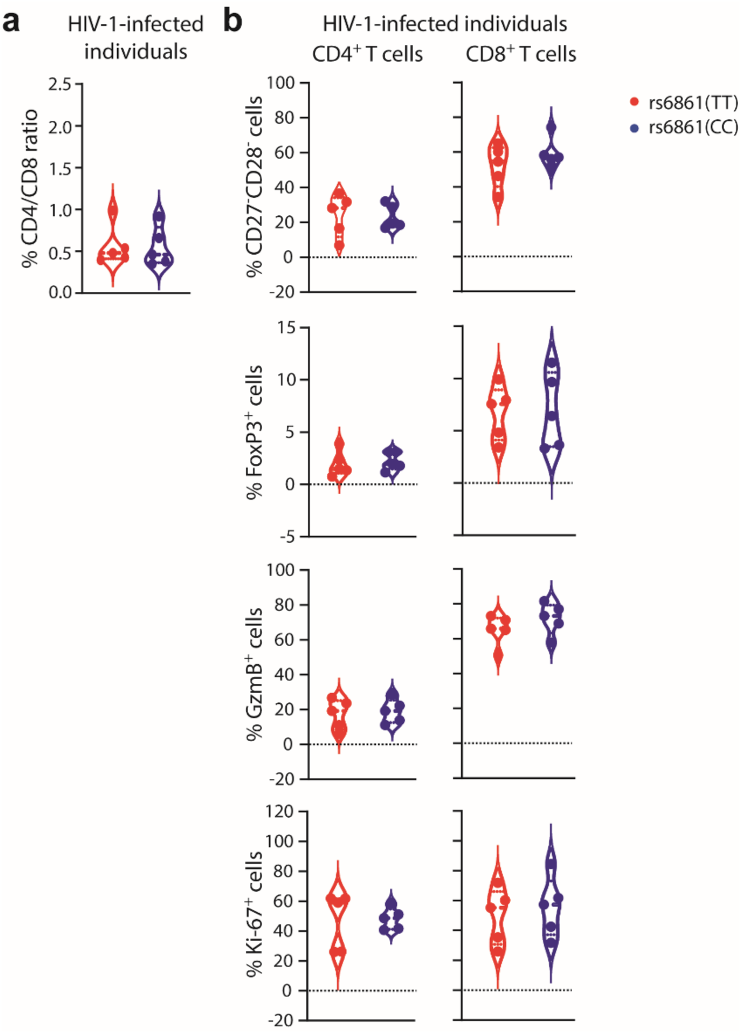

Supplementary Fig. 6. CD4/CD8 ratio and T cell features in HIV-1-infected individuals. Flow-cytometric analysis indicates **a.** CD4/CD8 ratio <1 in both rs6861(TT) and rs6861(CC) genotyped HIV-1-infected individuals (both n=5) as well as **b.** similar frequencies of CD27-CD28-, GzmB+, and Ki-67+ CD4+ and CD8+ T cells at steady-state. Frequencies also did not differ after T-cell activation (data not shown). Statistical significance was determined by independent two-tailed *t*-tests. Source data are provided as a Supplementary Source Data file.

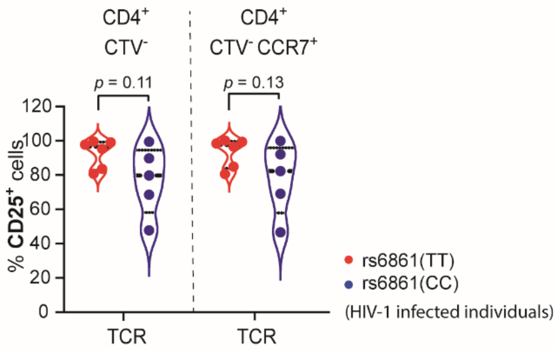

Supplementary Fig. 7. Frequency of CD25+ CD4+ T cells in rs6861(TT) HIV-1-infected individuals. The frequency of CD25+ CTV- and CTV-CCR7+ CD4+ T cells in rs6861(TT) (n=6) compared to rs6861(CC) (n=5) genotyped HIV-1-infected individuals upon TCR-engagement (independent two-tailed *t*-tests). Source data are provided as a Supplementary Source Data file.

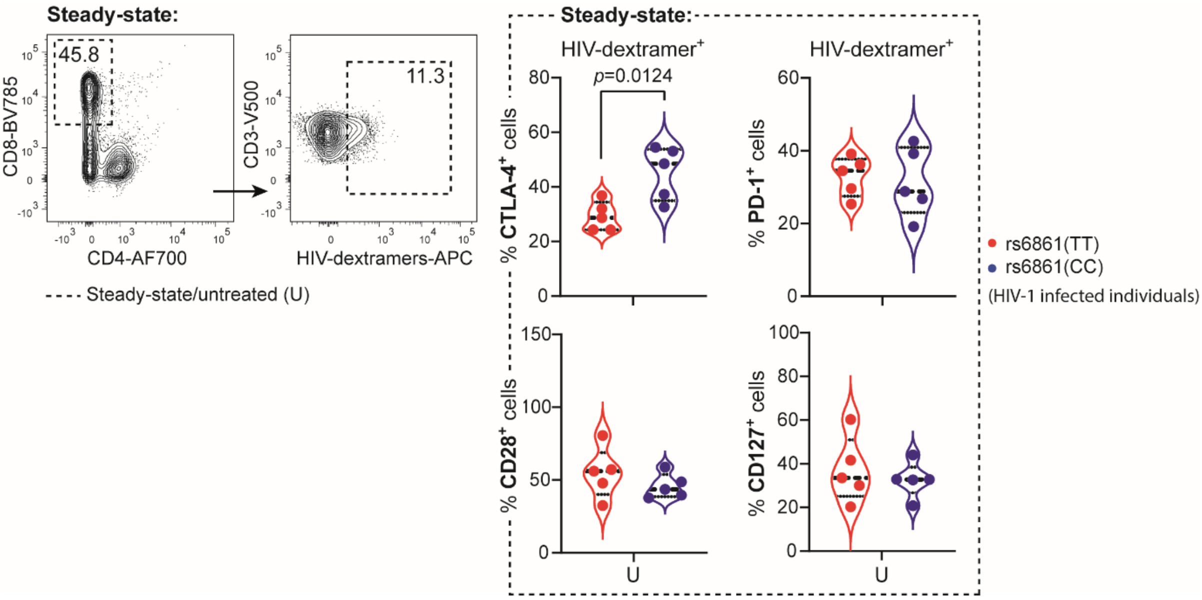

Supplementary Fig. 8. Increased frequency of CTLA-4+ HIV-1-specific CD8+ T cells in rs6861(CC) genotyped HIV-1-infected individuals. Flow-cytometric analysis of HIV-specific (MHC Class-I HIV-dextramer+) CD8+ T cells from genotyped HIV-1 infected individuals indicates decreased frequency of CTLA-4+ cells in rs6861(TT) compared to rs6861(CC) genotyped individuals (both n=5) and similar frequencies of PD-1+, CD28+, or CD127+ cells (independent two-tailed *t*-tests). U = untreated. See Fig. 6. Source data are provided as a Supplementary Source Data file.

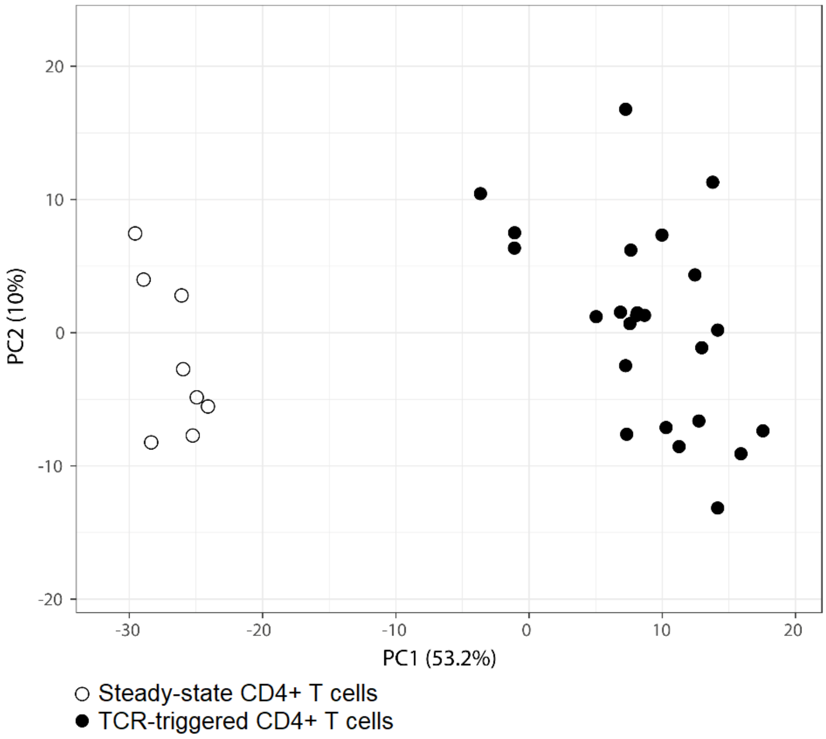

Supplementary Fig. 9. Principal component analysis reveals clear separation of steady-state and TCR-triggered CD4+ T cells. Analysis was performed using ClustVis[101] using count per million of the 500 genes with the highest variance with unit variance scaling. RNA-seq. data are accessible through GEO Series accession number GSE253769.

Gene	*p*-val	*p*-adj	Log2FC	Description
Steady-state CD4+ T cells				
IGLL5	1.65E-05	0.064	2.380	Immunoglobulin lambda-like polypeptide 5
IL17F	8.00E-05	0.187	-1.311	Interleukin 17F
OR11G2	2.46E-04	0.480	5.092	Olfactory receptor 11G2
SAR1A	3.11E-04	0.519	-0.776	Secretion associated Ras related GTPase 1A
IL1RN	4.13E-04	0.536	-1.765	Interleukin 1 receptor antagonist
YDJC	8.86E-04	0.671	0.946	Carbohydrate deacetylase
TIPARP	9.06E-04	0.671	0.940	TCDD inducible oly(ADP-ribose) polymerase
TRIM59	9.84E-04	0.671	-0.730	Tripartite motif containing 59
Activated CD4+ T cells				
IL17F	5.48E-06	0.021	-1.387	Interleukin 17F
HLA-DRB5	3.41E-05	0.053	4.190	Major histocompatibility complex Class II DR β5
CCL7	6.35E-04	0.544	1.897	Monocyte chemoattractant protein
LRRN3	7.38E-04	0.569	1.600	Leucin rich repeat neuronal 3
KPNA5	8.87E-04	0.612	1.166	Karyopherin subunit α5
IGLL5	9.56E-04	0.612	2.115	Immunoglobulin lambda-like polypeptide 5

Supplementary Table 2. Differentially expressed genes (DEGs) in genotyped CD4+ T cells. NGS RNA-seq. of FACSorted genotyped CD4+ T cells from healthy individuals at steady-state (untreated), or upon TCR-mediated activation with soluble anti-CD3 and anti-CD28 in combination with IL-7. Only DEGs (corrected for multiple testing using the FDR/Benjamini-Hochberg method) with unadjusted *p*-value<0.001 and max group mean >15 were included in the table (both adjusted and unadjusted *p*-values are shown), comparing gene expression in rs6861(TT) versus rs6861(CC) CD4+ T cells. Log2FC>0 represents upregulated gene expression and <0 represents suppressed gene expression in rs6861(TT) CD4+ T cells with rs6861(CC) CD4+ T cells set as a reasonable baseline. DEGs on X and Y chromosomes were excluded. *p*-val = unadjusted *p*-value, *p*-adj = FDR-adjusted *p*-value, Log2FC = log2 fold change. See Fig. 7. RNA-seq. data are accessible through GEO Series accession number GSE253769.

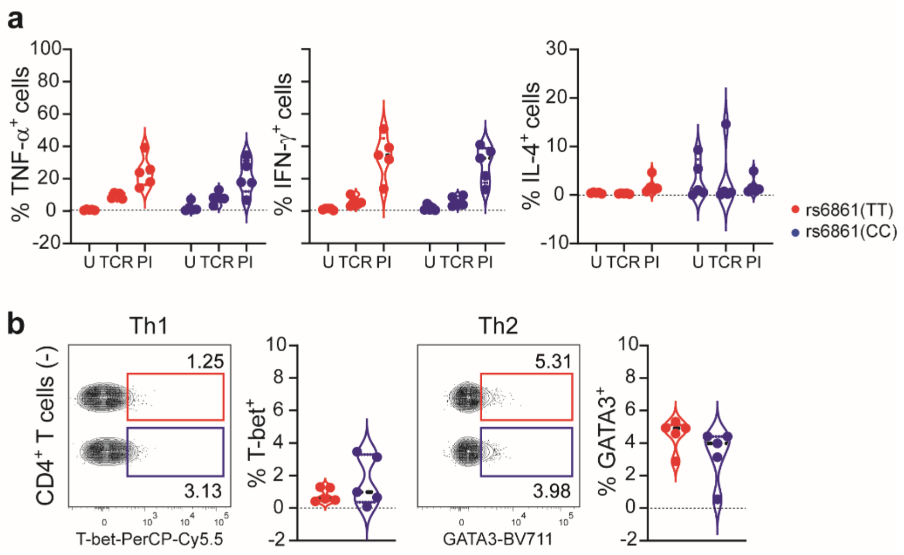

Supplementary Fig. 10. Similar frequency of Th1 and Th2 cells between genotyped individuals. a, b Intracellular flow cytometry data demonstrating similar frequencies of TNF-α+, IFN-γ+ (Th1), and IL-4+ (Th2) cells between rs6861(TT) versus rs6861(CC) genotyped healthy individuals (a), which is further corroborated by similar frequencies of genotyped CD4+ T cells expressing the hallmark Th transcription factors T-bet (Th1) and GATA3 (Th2) (b) (a,b n=5 per genotype). U = steady-state, untreated, TCR = anti-CD3/CD28, PI = PMA/Ionomycin. See Fig. 7. Source data are provided as a Supplementary Source Data file.

Antibody (clone)	Dilution	Source	Identifiers
Anti-CD45-BV711 (HI30)	1:100	BioLegend	Cat# 304049; RRID: AB_2563465
Anti-CD3-V500 (UCHT1)	1:50	BD Horizon	Cat# 561417; RRID: AB_10611584
Anti-CD3-APC-FIRE750 (UCHT1)	1:50	BioLegend	Cat# 300470; RRID: AB_2629689
Anti-CD4-PerCP-eFluor710 (SK3)	1:100	eBioscience	Cat# 46-0047; RRID: AB_1834402
Anti-CD4-AF488 (11830)	1:100	R&D Systems	Cat# FAB3791G
Anti-CD4-AF700 (SK3)	1:100	BioLegend	Cat# 344621; RRID: AB_2563149
Anti-CD8-BV785 (RPA-T8)	1:100	BioLegend	Cat# 301045; RRID: AB_11219195
Anti-CD8-PerCP-Cy5.5 (RPA-T8)	1:40	eBioscience	Cat# 45-0088; RRID: AB_1582255
Anti-CCR7-BUVB395 (3D12)	1:50	BD OptiBuild	Cat# 740267; RRID: AB_2740009
Anti-CD45RA-BV650 (HI100)	1:400	BD Horizon	Cat# 563963; RRID: AB_2738514
Anti-CD25-BUV737 (2A3)	1:100	BD Horizon	Cat# 612807; RRID: AB_2916878
Anti-CD127-PE-Cy7 (eBioRDR5)	1:100	eBioscience	Cat# 25-1278; RRID: AB_1659675
Anti-CTLA-4-BV711 (BNI3)	1:25	BioLegend	Cat# 369631; RRID: AB_2892450
Anti-PD-1-APC-Cy7 (EH12.2H7)	1:50	BioLegend	Cat# 329921; RRID: AB_10900982
Anti-PD-1-BV785 (EH12.2H7)	1:50	BioLegend	Cat# 329929; RRID: AB_11218984
Anti-CD27-BUV737 (L128)	1:50	BD Horizon	Cat# 612830; RRID: AB_2744350
Anti-CD27-BV711 (O323)	1:200	BioLegend	Cat# 302833; RRID: AB_11219201
Anti-CD28-FITC (CD28.2)	1:50	BioLegend	Cat# 302906; RRID: AB_314308
Anti-CD28-PE (CD28.2)	1:100	eBioscience	Cat# 12-0289; RRID: AB_2016668
Anti-CD137-PerCP-Cy5.5 (4-1BB)	1:50	BioLegend	Cat# 309813; RRID: AB_2205687
Anti-GzmB-AF700 (GB11)	1:160	BD Biosciences	Cat# 561016; RRID: AB_2033973
Anti-Ki-67-BV711 (Ki-67)	1:40	BioLegend	Cat# 350515; RRID: AB_11218996
Anti-IL-17F-AF488 (Poly5166)	1:100	BioLegend	Cat# 516603; RRID: AB_10730721
Anti-IL-17A-PE (BL168)	1:100	BioLegend	Cat# 512305; RRID: AB_961395
Anti-IL-22-APC-FIRE750 (2G12A41)	1:30	BioLegend	Cat# 366713; RRID: AB_2734410
Anti-IL-10-BV421 (JES3-9D7)	1:30	BD Horizon	Cat# 566276; RRID: AB_2738566
Anti-TNF-α-BUV395 (MAb11)	1:100	BD Horizon	Cat# 563996; RRID: AB_2738533
Anti-IFN-γ-BUV737 (4S.B3)	1:100	BD Horizon	Cat# 612845; RRID: AB_2869591
Anti-IL-4-PE-Cy7 (MP4-25D2)	1:100	BioLegend	Cat# 500823; RRID: AB_2126747
Anti-RORγτ-BV650 (Q21-559)	1:40	BD Horizon	Cat# 563424; RRID: AB_2738197
Anti-FoxP3-eFluor660 (PCH101)	1:40	eBioscience	Cat# 606-4776; RRID: AB_2896276
Anti-T-bet-PerCP-Cy5.5 (4B10)	1:100	BioLegend	Cat# 644805; RRID: AB_1595593
Anti-GATA3 (L50-823)	1:100	BD Horizon	Cat# 565449; RRID: AB_2739242
Anti-TRIM5α-AF594 (D-6)	1:200	Santa-Cruz	Cat# sc-373864; RRID: AB_10918111
Anti-LC3, unconjugated (4E12)	See legend	MBL Life science	Cat# M152-3; RRID: AB_1279144
Anti-HIV GAG A*0201/SLYNTVATL-APC (HLA-A*0201)	10 uL/3 million PBMC (see legend)	Immudex	Cat# WB2194
Anti-HIV GAG B*0702/GPGHKARVL-APC (HLA-B*0702)	10 uL/3 million PBMC (see legend)	Immudex	Cat# WH3590

Supplementary Table 3. List of commercial antibodies used in this study. Each antibody was validated and titrated before use and its optimal dilution as used in this study is given. Anti-LC3 (4E12) is routinely used unconjugated in a 1:20 dilution, here we conjugated Anti-LC3 (4E12) to AF488 using a Lightning-Link kit (Expedeon) and used it in a 1:200 dilution. Anti-HIV GAG-APC (Immudex) dextramers were used according to manufacturer instructions at 10 uL per 3 million PBMC. Cat# = catalogue number, RRID = Research resource identifier.
