# Supplementary material for: Autophagy-enhancing ATG16L1 polymorphism is associated with improved clinical outcome and T-cell immunity in chronic HIV-1 infection
Source: Nat Commun. 2024 Mar 28;15:2465. doi: 10.1038/s41467-024-46606-z (PMC10979031; doi:10.1038/s41467-024-46606-z)
Supplement: Supplementary file 3 — Reporting Summary [file 41467_2024_46606_MOESM3_ESM.pdf]

nature portfolio

Corresponding author(s): Dr. Carla Ribeiro

Last updated by author(s): 13/02/2024

Reporting Summary

Nature Portfolio wishes to improve the reproducibility of the work that we publish. This form provides structure for consistency and transparency in reporting. For further information on Nature Portfolio policies, see our Editorial Policies and the Editorial Policy Checklist.

Statistics

For all statistical analyses, confirm that the following items are present in the figure legend, table legend, main text, or Methods section.

n/a	Confirmed	
☐	☒	The exact sample size (*n*) for each experimental group/condition, given as a discrete number and unit of measurement
☐	☒	A statement on whether measurements were taken from distinct samples or whether the same sample was measured repeatedly
☐	☒	The statistical test(s) used AND whether they are one- or two-sided<br>*Only common tests should be described solely by name; describe more complex techniques in the Methods section.*
☒	☐	A description of all covariates tested
☐	☒	A description of any assumptions or corrections, such as tests of normality and adjustment for multiple comparisons
☐	☒	A full description of the statistical parameters including central tendency (e.g. means) or other basic estimates (e.g. regression coefficient) AND variation (e.g. standard deviation) or associated estimates of uncertainty (e.g. confidence intervals)
☐	☒	For null hypothesis testing, the test statistic (e.g. *F*, *t*, *r*) with confidence intervals, effect sizes, degrees of freedom and *P* value noted<br>*Give P values as exact values whenever suitable.*
☒	☐	For Bayesian analysis, information on the choice of priors and Markov chain Monte Carlo settings
☒	☐	For hierarchical and complex designs, identification of the appropriate level for tests and full reporting of outcomes
☒	☐	Estimates of effect sizes (e.g. Cohen's *d*, Pearson's *r*), indicating how they were calculated

Our web collection on statistics for biologists contains articles on many of the points above.

Software and code

Policy information about availability of computer code

Data collection	Microsoft office Excel 2016
Data analysis	Microsoft office Excel 2016, Graphpad Prism (version 9), FACSDIVA (version 10.8.1), Qiagen's CLC Genomics Workbench (21.0.1), clusterProfiler (PMID22455463), EdgeR (PMID19910308), MORPHEUS (https://software.broadinstitute.org/morpheus), ClustVis (PMID25969447), GO Biological Process (c5.go.bp.v7.4)

For manuscripts utilizing custom algorithms or software that are central to the research but not yet described in published literature, software must be made available to editors and reviewers. We strongly encourage code deposition in a community repository (e.g. GitHub). See the Nature Portfolio guidelines for submitting code & software for further information.

Data

Policy information about availability of data

All manuscripts must include a data availability statement. This statement should provide the following information, where applicable:

- Accession codes, unique identifiers, or web links for publicly available datasets
- A description of any restrictions on data availability
- For clinical datasets or third party data, please ensure that the statement adheres to our policy

The authors declare that the data supporting the findings of this study are available within this paper and its source data files. The RNA seq data discussed in this publication have been deposited in NCBI's Gene Expression Omnibus (Edgar et al., 2002) and are accessible through GEO Series accession number GSE253769

April 2023

(https://www.ncbi.nlm.nih.gov/geo/query/acc.cgi?acc=GSE253769). This article does not contain any original code. Requests for data should be made to and will be fulfilled by C.M.S. Ribeiro (c.m.ribeiro@amsterdamumc.nl), provided the data will be used within the scope of the originally provided informed consent.

Research involving human participants, their data, or biological material

Policy information about studies with human participants or human data. See also policy information about sex, gender (identity/presentation), and sexual orientation and race, ethnicity and racism.

Reporting on sex and gender	Biological sex of the participants has been specified where appropriate. Gender was not addressed.
Reporting on race, ethnicity, or other socially relevant groupings	N/A
Population characteristics	The Amsterdam Cohort Studies on HIV infection and AIDS is a prospective study among men who have sex with men (MSM) that started in 1984. The HIV-1-infected MSM were enrolled in the cohort between October 1984 and March 1986, were actively followed-up until May 1996 and none of the 304 genotyped MSM received effective ART before AIDS diagnosis as this was not available at the time. From these 304 genotyped MSM, PBMCs from n=6 rs6861(TT) and n=6 rs6861(CC) SNP were selected. These 12 individuals were also matched based on coviariate-relevant population characteristics sex (male), seroconversion year, age at seroconversion (between 25-38 years old), time between seroconversion and PBMC collection, time between PBMC collection and AIDS diagnosis or AIDS-free follow-up, CD4 count (>400/mL), and absence of confounding SNPs CCR5d32, HLA-B27, HLA-B57, and ATG16L1-T300A. Exact age of the individual participants was not disclosed.<br><br>PBMCs isolated from buffy coats derived from blood from healthy individuals supplied for research purposes by Sanquin Blood Supply Foundation (Amsterdam) were included in this study. Individual characteristics of these donors (such as sex, age, etc.) are not disclosed.
Recruitment	Data and material from HIV-1-infected individuals was collected during a previous study (PMID:21811574). Recruitment of volunteers for the Amsterdam Cohort Studies on HIV infection and AIDS started in 1984 and was performed by the Municipal Health Service Amsterdam. Recruited volunteers were all men who had sex with men in the six months prior to recruitment and lived mainly around the city of Amsterdam, The Netherlands. Recruitment was limited to men in the age range of 18 to 30 years old and was done through outreach activities at MSM meeting places, online adverstisement, and participants recruiting participants.<br><br>Healthy blood donors were recruited by Sanquin Blood Supply Foundation (Amsterdam) for research purposes and was approved by the Ethics Advisory Body of the Sanquin Blood Supply Foundation (Amsterdam, the Netherlands).
Ethics oversight	This study has been conducted in accordance with the ethical principles set out in the declaration of Helsinki. The study was approved by the Amsterdam UMC institutional Medical Ethics Review Committee of the University of Amsterdam (Amsterdam, the Netherlands), the Ethics Advisory Body of the Sanquin Blood Supply Foundation (Amsterdam, the Netherlands), and the board of the Amsterdam Cohort Studies (ACS) on HIV infection and AIDS (Amsterdam, the Netherlands). Written consent was obtained from ACS study participants. Use of buffy coats from healthy blood donors is not subjected to informed consent according to the Medical Research Involving Human Subjects Act and the Medical Ethics Review Committee of the Amsterdam UMC. All methods were performed in accordance with the relevant guidelines and regulations as stated in the Amsterdam UMC Research Code.

Note that full information on the approval of the study protocol must also be provided in the manuscript.

Field-specific reporting

Please select the one below that is the best fit for your research. If you are not sure, read the appropriate sections before making your selection.

[x] Life sciences [ ] Behavioural & social sciences [ ] Ecological, evolutionary & environmental sciences

For a reference copy of the document with all sections, see nature.com/documents/nr-reporting-summary-flat.pdf

Life sciences study design

All studies must disclose on these points even when the disclosure is negative.

Sample size	No statistical methods were used to predetermine sample size. Sample size were chosen based on the limited resources matching the genotyping and clinical characteristics criteria and with an expected effect size based on previous research and related literature.
Data exclusions	PBMC data from 1 HIV-1-infected rs6861(CC) individual were discarded due to insufficient viable cells in the sample. For RNA-seq. data, buffy coats from healthy individuals was screened for ATG16L1 T300A rs2241880 SNP (TaqMan Sample-to-SNP kit, ThermoFisher) and homozygous minor donors for ATG16L1 T300A donors were excluded from RNA-seq. experiments. PBMC samples from HIV-infected ACS participants homozygous minor for this ATG16L1 T300A were also excluded from flow cytometry analyses.
Replication	Typically, experiments were replicated at least once to confirm robustness of biological observations.

Randomization	Randomization was not relevant to the clinical analyses because all data of the seroconverter follow up cohort was used. Functional analyses were performed on biological materials matching the variables relevant to the aim of the study: rs6861 (TT vs CC) genotype, year of seroconversion,CD4 count, time between seroconversion, sample selecton and AIDS diagnosis.
Blinding	Experiments were not blinded but kept as unbiased as possible and results were always confirmed by at least two scientists.

Behavioural & social sciences study design

All studies must disclose on these points even when the disclosure is negative.

Study description	*Briefly describe the study type including whether data are quantitative, qualitative, or mixed-methods (e.g. qualitative cross-sectional, quantitative experimental, mixed-methods case study).*
Research sample	*State the research sample (e.g. Harvard university undergraduates, villagers in rural India) and provide relevant demographic information (e.g. age, sex) and indicate whether the sample is representative. Provide a rationale for the study sample chosen. For studies involving existing datasets, please describe the dataset and source.*
Sampling strategy	*Describe the sampling procedure (e.g. random, snowball, stratified, convenience). Describe the statistical methods that were used to predetermine sample size OR if no sample-size calculation was performed, describe how sample sizes were chosen and provide a rationale for why these sample sizes are sufficient. For qualitative data, please indicate whether data saturation was considered, and what criteria were used to decide that no further sampling was needed.*
Data collection	*Provide details about the data collection procedure, including the instruments or devices used to record the data (e.g. pen and paper, computer, eye tracker, video or audio equipment) whether anyone was present besides the participant(s) and the researcher, and whether the researcher was blind to experimental condition and/or the study hypothesis during data collection.*
Timing	*Indicate the start and stop dates of data collection. If there is a gap between collection periods, state the dates for each sample cohort.*
Data exclusions	*If no data were excluded from the analyses, state so OR if data were excluded, provide the exact number of exclusions and the rationale behind them, indicating whether exclusion criteria were pre-established.*
Non-participation	*State how many participants dropped out/declined participation and the reason(s) given OR provide response rate OR state that no participants dropped out/declined participation.*
Randomization	*If participants were not allocated into experimental groups, state so OR describe how participants were allocated to groups, and if allocation was not random, describe how covariates were controlled.*

Ecological, evolutionary & environmental sciences study design

All studies must disclose on these points even when the disclosure is negative.

Study description	*Briefly describe the study. For quantitative data include treatment factors and interactions, design structure (e.g. factorial, nested, hierarchical), nature and number of experimental units and replicates.*
Research sample	*Describe the research sample (e.g. a group of tagged Passer domesticus, all Stenocereus thurberi within Organ Pipe Cactus National Monument), and provide a rationale for the sample choice. When relevant, describe the organism taxa, source, sex, age range and any manipulations. State what population the sample is meant to represent when applicable. For studies involving existing datasets, describe the data and its source.*
Sampling strategy	*Note the sampling procedure. Describe the statistical methods that were used to predetermine sample size OR if no sample-size calculation was performed, describe how sample sizes were chosen and provide a rationale for why these sample sizes are sufficient.*
Data collection	*Describe the data collection procedure, including who recorded the data and how.*
Timing and spatial scale	*Indicate the start and stop dates of data collection, noting the frequency and periodicity of sampling and providing a rationale for these choices. If there is a gap between collection periods, state the dates for each sample cohort. Specify the spatial scale from which the data are taken*
Data exclusions	*If no data were excluded from the analyses, state so OR if data were excluded, describe the exclusions and the rationale behind them, indicating whether exclusion criteria were pre-established.*
Reproducibility	*Describe the measures taken to verify the reproducibility of experimental findings. For each experiment, note whether any attempts to repeat the experiment failed OR state that all attempts to repeat the experiment were successful.*
Randomization	*Describe how samples/organisms/participants were allocated into groups. If allocation was not random, describe how covariates were controlled. If this is not relevant to your study, explain why.*

Blinding

Describe the extent of blinding used during data acquisition and analysis. If blinding was not possible, describe why OR explain why blinding was not relevant to your study.

Did the study involve field work? [ ] Yes [ ] No

Field work, collection and transport

Field conditions

Describe the study conditions for field work, providing relevant parameters (e.g. temperature, rainfall).

Location

State the location of the sampling or experiment, providing relevant parameters (e.g. latitude and longitude, elevation, water depth).

Access & import/export

Describe the efforts you have made to access habitats and to collect and import/export your samples in a responsible manner and in compliance with local, national and international laws, noting any permits that were obtained (give the name of the issuing authority, the date of issue, and any identifying information).

Disturbance

Describe any disturbance caused by the study and how it was minimized.

Reporting for specific materials, systems and methods

We require information from authors about some types of materials, experimental systems and methods used in many studies. Here, indicate whether each material, system or method listed is relevant to your study. If you are not sure if a list item applies to your research, read the appropriate section before selecting a response.

Materials & experimental systems

n/a	Involved in the study
[ ]	[x] Antibodies
[ ]	[x] Eukaryotic cell lines
[x]	[ ] Palaeontology and archaeology
[x]	[ ] Animals and other organisms
[x]	[ ] Clinical data
[x]	[ ] Dual use research of concern
[x]	[ ] Plants

Methods

n/a	Involved in the study
[x]	[ ] ChIP-seq
[ ]	[x] Flow cytometry
[x]	[ ] MRI-based neuroimaging

Antibodies

Antibodies used

Anti-CD45-BV711 (HI30) 1:100 BioLegend Cat# 304049; RRID: AB_2563465
Anti-CD3-V500 (UCHT1) 1:50 BD Horizon Cat# 561417; RRID: AB_10611584
Anti-CD3-APC-FIRE750 (UCHT1) 1:50 BioLegend Cat# 300470; RRID: AB_2629689
Anti-CD4-PerCP-eFluor710 (SK3) 1:100 eBioscience Cat# 46-0047; RRID: AB_1834402
Anti-CD4-AF488 (11830) 1:100 R&D Systems Cat# FAB3791G
Anti-CD4-AF700 (SK3)1:100 BioLegend Cat# 344621; RRID: AB_2563149
Anti-CD8-BV785 (RPA-T8) 1:100 BioLegend Cat# 301045; RRID: AB_11219195
Anti-CD8-PerCP-Cy5.5 (RPA-T8) 1:40 eBioscience Cat# 45-0088; RRID: AB_1582255
Anti-CCR7-BUVB395 (3D12) 1:50 BD OptiBuild Cat# 740267; RRID: AB_2740009
Anti-CD45RA-BV650 (HI100) 1:400 BD Horizon Cat# 563963; RRID: AB_2738514
Anti-CD25-BUV737 (2A3) 1:100 BD Horizon Cat# 612807; RRID: AB_2916878
Anti-CD127-PE-Cy7 (eBioRDR5) 1:100 eBioscience Cat# 25-1278; RRID: AB_1659675
Anti-CTLA-4-BV711 (BNI3) 1:25 BioLegend Cat# 369631; RRID: AB_2892450
Anti-PD-1-APC-Cy7 (EH12.2H7) 1:50 BioLegend Cat# 329921; RRID: AB_10900982
Anti-PD-1-BV785 (EH12.2H7) 1:50 BioLegend Cat# 329929; RRID: AB_11218984
Anti-CD27-BUV737 (L128) 1:50 BD Horizon Cat# 612830; RRID: AB_2744350
Anti-CD27-BV711 (O323) 1:200 BioLegend Cat# 302833; RRID: AB_11219201
Anti-CD28-FITC (CD28.2) 1:50 BioLegend Cat# 302906; RRID: AB_314308
Anti-CD28-PE (CD28.2) 1:100 eBioscience Cat# 12-0289; RRID: AB_2016668
Anti-CD137-PerCP-Cy5.5 (4-1BB) 1:50 BioLegend Cat# 309813; RRID: AB_2205687
Anti-GzmB-AF700 (GB11) 1:160 BD Biosciences Cat# 561016; RRID: AB_2033973
Anti-Ki-67-BV711 (Ki-67) 1:40 BioLegend Cat# 350515; RRID: AB_11218996
Anti-IL-17F-AF488 (Poly5166) 1:100 BioLegend Cat# 516603; RRID: AB_10730721
Anti-IL-17A-PE (BL168) 1:100 BioLegend Cat# 512305; RRID: AB_961395
Anti-IL-22-APC-FIRE750 (2G12A41)1:30 BioLegend Cat# 366713; RRID: AB_2734410
Anti-IL-10-BV421 (JES3-9D7) 1:30 BD Horizon Cat# 566276; RRID: AB_2738566

Anti-TNF-α-BUV395 (MAb11)	1:100	BD Horizon	Cat# 563996; RRID: AB_2738533
Anti-IFN-γ-BUV737 (4S.B3)	1:100	BD Horizon	Cat# 612845; RRID: AB_2869591
Anti-IL-4-PE-Cy7 (MP4-25D2)	1:100	BioLegend	Cat# 500823; RRID: AB_2126747
Anti-RORγτ-BV650 (Q21-559)	1:40	BD Horizon	Cat# 563424; RRID: AB_2738197
Anti-FoxP3-eFluor660 (PCH101)	1:40	eBioscience	Cat# 606-4776; RRID: AB_2896276
Anti-T-bet-PerCP-Cy5.5 (4B10)	1:100	BioLegend	Cat# 644805; RRID: AB_1595593
Anti-GATA3 (L50-823)1:100		BD Horizon	Cat# 565449; RRID: AB_2739242
Anti-TRIM5α-AF594 (D-6)	1:200	Santa-Cruz	Cat# sc-373864; RRID: AB_10918111
Anti-LC3 (4E12) conjugated to AF488	1:200	MBL Life science	Cat# M152-3; RRID: AB_1279144
Anti-HIV GAG A*0201/SLYNTVATL-APC (HLA-A*0201)	10 uL per 3 million PBMC	Immudex	Cat# WB2194
Anti-HIV GAG B*0702/GPGHKARVL-APC (HLA-B*0702)	10 uL per 3 million PBMC	Immudex	Cat# WH3590

Validation

Each antibody was validated and titrated in house prior to use by performing a titration series including negative (fluorescence minus one) controls. The following dilution were chosen:
Anti-CD45-BV711 (HI30) 1:100
Anti-CD3-V500 (UCHT1) 1:25
Anti-CD3-APC-FIRE750 (UCHT1) 1:50
Anti-CD4-PerCP-eFluor710 (SK3) 1:100
Anti-CD4-AF488 (11830) 1:100
Anti-CD4-AF700 (SK3) 1:100
Anti-CD8-BV785 (RPA-T8) 1:100
Anti-CD8-PerCP-Cy5.5 (RPA-T8) 1:40
Anti-CCR7-BUVB395 (3D12) 1:50
Anti-CD45RA-BV650 (HI100) 1:400
Anti-CD25-BUV737 (2A3) 1:100
Anti-CD127-PE-Cy7 (eBioRDR5) 1:100
Anti-CTLA-4-BV711 (BNI3) 1:25
Anti-PD-1-APC-Cy7 (EH12.2H7) 1:50
Anti-PD-1-BV785 (EH12.2H7) 1:50
Anti-CD27-BUV737 (L128) 1:50
Anti-CD27-BV711 (O323) 1:200
Anti-CD28-FITC (CD28.2) 1:50
Anti-CD28-PE (CD28.2) 1:100
Anti-CD137-PerCP-Cy5.5 (4-1BB) 1:50
Anti-GzmB-AF700 (GB11) 1:160
Anti-Ki-67-BV711 (Ki-67) 1:40
Anti-IL-17F-AF488 (Poly5166) 1:100
Anti-IL-17A-PE (BL168) 1:100
Anti-IL-22-APC-FIRE750 (2G12A41) 1:30
Anti-IL-10-BV421 (JES3-9D7) 1:30
Anti-TNF-α-BUV395 (MAb11) 1:100
Anti-IFN-γ-BUV737 (4S.B3) 1:100
Anti-IL-4-PE-Cy7 (MP4-25D2) 1:100
Anti-RORγτ-BV650 (Q21-559) 1:40
Anti-FoxP3-eFluor660 (PCH101) 1:40
Anti-T-bet-PerCP-Cy5.5 (4B10) 1:100
Anti-GATA3 (L50-823) 1:100
Anti-TRIM5α-AF594 (D-6) 1:200
Anti-LC3 (4E12) conjugated to AF488 (Lightning-link kit) 1:200
Validation based on the information provided by the manufacturer:
Anti-HIV GAG A*0201/SLYNTVATL-APC (HLA-A*0201) 10 uL per 3 million PBMC per manufacturer instructions - Immudex Cat# WB2194
Anti-HIV GAG B*0702/GPGHKARVL-APC (HLA-B*0702) 10 uL per 3 million PBMC per manufacturer instructions Immudex Cat# WH3590

Eukaryotic cell lines

Policy information about cell lines and Sex and Gender in Research

Cell line source(s)

Autophagy flux reporter cells were generated via retroviral transduction of U87.CD4.CCR5 cells (obtained via the NIH AIDS Reagent Program, Division of AIDS, NIAID, NIH repository) with pBABE-mCherry-GFP-LC3 (Addgene 22418).

Authentication

This cell line was not authenticated.

Mycoplasma contamination

The cell line was tested negative for mycoplasma contamination.

Commonly misidentified lines
(See ICLAC register)

No misidentified cell line listed in ICLA database was used.

Palaeontology and Archaeology

Specimen provenance	*Provide provenance information for specimens and describe permits that were obtained for the work (including the name of the issuing authority, the date of issue, and any identifying information). Permits should encompass collection and, where applicable, export.*
Specimen deposition	*Indicate where the specimens have been deposited to permit free access by other researchers.*
Dating methods	*If new dates are provided, describe how they were obtained (e.g. collection, storage, sample pretreatment and measurement), where they were obtained (i.e. lab name), the calibration program and the protocol for quality assurance OR state that no new dates are provided.*

☐ Tick this box to confirm that the raw and calibrated dates are available in the paper or in Supplementary Information.

Ethics oversight	*Identify the organization(s) that approved or provided guidance on the study protocol, OR state that no ethical approval or guidance was required and explain why not.*

Note that full information on the approval of the study protocol must also be provided in the manuscript.

Animals and other research organisms

Policy information about studies involving animals; ARRIVE guidelines recommended for reporting animal research, and Sex and Gender in Research

Laboratory animals	*For laboratory animals, report species, strain and age OR state that the study did not involve laboratory animals.*
Wild animals	*Provide details on animals observed in or captured in the field; report species and age where possible. Describe how animals were caught and transported and what happened to captive animals after the study (if killed, explain why and describe method; if released, say where and when) OR state that the study did not involve wild animals.*
Reporting on sex	*Indicate if findings apply to only one sex; describe whether sex was considered in study design, methods used for assigning sex. Provide data disaggregated for sex where this information has been collected in the source data as appropriate; provide overall numbers in this Reporting Summary. Please state if this information has not been collected. Report sex-based analyses where performed, justify reasons for lack of sex-based analysis.*
Field-collected samples	*For laboratory work with field-collected samples, describe all relevant parameters such as housing, maintenance, temperature, photoperiod and end-of-experiment protocol OR state that the study did not involve samples collected from the field.*
Ethics oversight	*Identify the organization(s) that approved or provided guidance on the study protocol, OR state that no ethical approval or guidance was required and explain why not.*

Note that full information on the approval of the study protocol must also be provided in the manuscript.

Clinical data

Policy information about clinical studies

All manuscripts should comply with the ICMJEguidelines for publication of clinical research and a completedCONSORT checklist must be included with all submissions.

Clinical trial registration	*Provide the trial registration number from ClinicalTrials.gov or an equivalent agency.*
Study protocol	*Note where the full trial protocol can be accessed OR if not available, explain why.*
Data collection	*Describe the settings and locales of data collection, noting the time periods of recruitment and data collection.*
Outcomes	*Describe how you pre-defined primary and secondary outcome measures and how you assessed these measures.*

Dual use research of concern

Policy information about dual use research of concern

Hazards

Could the accidental, deliberate or reckless misuse of agents or technologies generated in the work, or the application of information presented in the manuscript, pose a threat to:

No	Yes	
☒	☐	Public health
☒	☐	National security
☒	☐	Crops and/or livestock
☒	☐	Ecosystems
☒	☐	Any other significant area

Experiments of concern

Does the work involve any of these experiments of concern:

No	Yes	
☒	☐	Demonstrate how to render a vaccine ineffective
☒	☐	Confer resistance to therapeutically useful antibiotics or antiviral agents
☒	☐	Enhance the virulence of a pathogen or render a nonpathogen virulent
☒	☐	Increase transmissibility of a pathogen
☒	☐	Alter the host range of a pathogen
☒	☐	Enable evasion of diagnostic/detection modalities
☒	☐	Enable the weaponization of a biological agent or toxin
☒	☐	Any other potentially harmful combination of experiments and agents

Plants

Seed stocks	*Report on the source of all seed stocks or other plant material used. If applicable, state the seed stock centre and catalogue number. If plant specimens were collected from the field, describe the collection location, date and sampling procedures.*
Novel plant genotypes	*Describe the methods by which all novel plant genotypes were produced. This includes those generated by transgenic approaches, gene editing, chemical/radiation-based mutagenesis and hybridization. For transgenic lines, describe the transformation method, the number of independent lines analyzed and the generation upon which experiments were performed. For gene-edited lines, describe the editor used, the endogenous sequence targeted for editing, the targeting guide RNA sequence (if applicable) and how the editor was applied.*
Authentication	*Describe any authentication procedures for each seed stock used or novel genotype generated. Describe any experiments used to assess the effect of a mutation and, where applicable, how potential secondary effects (e.g. second site T-DNA insertions, mosiacism, off-target gene editing) were examined.*

ChIP-seq

Data deposition

☐ Confirm that both raw and final processed data have been deposited in a public database such as GEO.

☐ Confirm that you have deposited or provided access to graph files (e.g. BED files) for the called peaks.

Data access links<br>*May remain private before publication.*	*For "Initial submission" or "Revised version" documents, provide reviewer access links. For your "Final submission" document, provide a link to the deposited data.*
Files in database submission	*Provide a list of all files available in the database submission.*
Genome browser session<br>(e.g. UCSC)	*Provide a link to an anonymized genome browser session for "Initial submission" and "Revised version" documents only, to enable peer review. Write "no longer applicable" for "Final submission" documents.*

Methodology

Replicates	*Describe the experimental replicates, specifying number, type and replicate agreement.*
Sequencing depth	*Describe the sequencing depth for each experiment, providing the total number of reads, uniquely mapped reads, length of reads and whether they were paired- or single-end.*
Antibodies	*Describe the antibodies used for the ChIP-seq experiments; as applicable, provide supplier name, catalog number, clone name, and lot number.*
Peak calling parameters	*Specify the command line program and parameters used for read mapping and peak calling, including the ChIP, control and index files used.*

Data quality

Describe the methods used to ensure data quality in full detail, including how many peaks are at FDR 5% and above 5-fold enrichment.

Software

Describe the software used to collect and analyze the ChIP-seq data. For custom code that has been deposited into a community repository, provide accession details.

Flow Cytometry

Plots

Confirm that:

☒ The axis labels state the marker and fluorochrome used (e.g. CD4-FITC).

☒ The axis scales are clearly visible. Include numbers along axes only for bottom left plot of group (a 'group' is an analysis of identical markers).

☒ All plots are contour plots with outliers or pseudocolor plots.

☒ A numerical value for number of cells or percentage (with statistics) is provided.

Methodology

Sample preparation

PBMCs were isolated from buffy coats derived from blood of healthy donors (Sanquin Blood Supply Foundation) via density gradient centrifugation and subsequently frozen until use. Blood donations are anonymized without access to information (such as age, sex, relatedness) pertaining the individual donors. In brief, diluted blood was layered on top of Lymphoprep (Axis-Shield) and centrifuged for 22 minutes at room temperature and 1000 g with no break after which the mononuclear cell fraction was aspirated, washed and frozen in buffer containing 10% dimethyl sulfoxide (DMSO; Amresco) and minimum 30% fetal bovine serum (FBS; Biological Industries) at -196°C. Frozen PBMC from HIV-1-infected individuals (ACS) and healthy donors (Sanquin Blood Supply Foundation) were thawed in buffer containing minimum 30% FBS and no antibiotics, washed and left to recover in the incubator (37°C and 5% CO2) in Roswell Park Memorial Institute (RPMI; Sigma-Aldrich) medium containing 10% FBS and 1% penicillin/streptomycin (P/S; Invitrogen), ‘T-cell medium’ used for all assays unless described otherwise, for a minimum of 16 hours to recover. Automated cell counting was used to determine the number of viable cells.

For surface-molecule staining, cells were incubated with a cocktail of titrated antibodies (see Supplementary Table 3 for a full list of antibodies) and LIVE/DEAD Fixable viability dye (ThermoFisher) diluted in PBS for 30 minutes, shielded from light, shaking at 600 strokes/minute at 4°C, washed twice, then fixated using 4% PFA (Electron Microscopy Sciences), washed and stored in PBS. HIV-specific CD8+ T cells were detected using MHC Dextramer technology according to the manufacturer’s instructions (Immudex); MHC Class I HIV GAG HLA-A*0201 (SLYNTVATL) and HIV GAG HLA-B*0702 (GPGHKARVL) based on human leukocyte antigen (HLA)-typing performed by the ACS, which indicated most selected HIV-1-infected individuals to be HLA-A2 (n = 6) or HLA-B7 (n = 6) positive. For intracellular staining (except for LC3-II, see next paragraph), surface-stained cells were washed and fixated using Fixation/Permeabilization reagent (FoxP3/Transcription Staining Buffer Set; eBioscience) for 15 minutes, shielded from light, shaking at 600 strokes/minute at 4°C, washed once, then stained intracellularly with a cocktail of titrated antibodies diluted in Permeabilization Buffer (FoxP3/Transcription Staining Buffer Set; eBioscience) for 30 minutes, shielded from light, shaking at 600 strokes/minute at 4°C, then washed twice and stored in PBS. Samples were acquired within 24 hours after staining.

Instrument

Fortessa Cell Analyzer (Becton Dickinson)

Software

FACSDIVA

Cell population abundance

Complete CD4+ and CD8+ T cell subsets with viability >70% were evaluated.

Gating strategy

In all flow-cytometry analyses, debris and dead cells were excluded based on forward- and sideward-scatter and LIVE/DEAD fixable viability dyes. Subsequently, lymphocytes were selected based on forward-and sideward-scatter and doublets were always removed based on FSC-H versus FSC-W and SSC-H versus SSC-W discrimination. CD4+ T cells were identified as CD3+CD4+CD8- (or as CD3+CD8- in the case of CD4-downregulation such as after stimulation) and CD8+ T cells were identified as CD3+CD8+CD4- cells. Details on further gating strategy and representative FACS plots of all analyzed T-cell subsets are provided in the main and supplementary figures where appropriate.

☒ Tick this box to confirm that a figure exemplifying the gating strategy is provided in the Supplementary Information.

Magnetic resonance imaging

Experimental design

Design type

Indicate task or resting state; event-related or block design.

Design specifications

Specify the number of blocks, trials or experimental units per session and/or subject, and specify the length of each trial or block (if trials are blocked) and interval between trials.

Behavioral performance measures

State number and/or type of variables recorded (e.g. correct button press, response time) and what statistics were used to establish that the subjects were performing the task as expected (e.g. mean, range, and/or standard deviation across subjects).

Acquisition

Imaging type(s) | *Specify: functional, structural, diffusion, perfusion.*

Field strength | *Specify in Tesla*

Sequence & imaging parameters | *Specify the pulse sequence type (gradient echo, spin echo, etc.), imaging type (EPI, spiral, etc.), field of view, matrix size, slice thickness, orientation and TE/TR/flip angle.*

Area of acquisition | *State whether a whole brain scan was used OR define the area of acquisition, describing how the region was determined.*

Diffusion MRI ☐ Used ☐ Not used

Preprocessing

Preprocessing software | *Provide detail on software version and revision number and on specific parameters (model/functions, brain extraction, segmentation, smoothing kernel size, etc.).*

Normalization | *If data were normalized/standardized, describe the approach(es): specify linear or non-linear and define image types used for transformation OR indicate that data were not normalized and explain rationale for lack of normalization.*

Normalization template | *Describe the template used for normalization/transformation, specifying subject space or group standardized space (e.g. original Talairach, MNI305, ICBM152) OR indicate that the data were not normalized.*

Noise and artifact removal | *Describe your procedure(s) for artifact and structured noise removal, specifying motion parameters, tissue signals and physiological signals (heart rate, respiration).*

Volume censoring | *Define your software and/or method and criteria for volume censoring, and state the extent of such censoring.*

Statistical modeling & inference

Model type and settings | *Specify type (mass univariate, multivariate, RSA, predictive, etc.) and describe essential details of the model at the first and second levels (e.g. fixed, random or mixed effects; drift or auto-correlation).*

Effect(s) tested | *Define precise effect in terms of the task or stimulus conditions instead of psychological concepts and indicate whether ANOVA or factorial designs were used.*

Specify type of analysis: ☐ Whole brain ☐ ROI-based ☐ Both

Statistic type for inference | *Specify voxel-wise or cluster-wise and report all relevant parameters for cluster-wise methods.*

(See Eklund et al. 2016)

Correction | *Describe the type of correction and how it is obtained for multiple comparisons (e.g. FWE, FDR, permutation or Monte Carlo).*

Models & analysis

n/a	Involved in the study
☐	☐ Functional and/or effective connectivity
☐	☐ Graph analysis
☐	☐ Multivariate modeling or predictive analysis

Functional and/or effective connectivity | *Report the measures of dependence used and the model details (e.g. Pearson correlation, partial correlation, mutual information).*

Graph analysis | *Report the dependent variable and connectivity measure, specifying weighted graph or binarized graph, subject- or group-level, and the global and/or node summaries used (e.g. clustering coefficient, efficiency, etc.).*

Multivariate modeling and predictive analysis | *Specify independent variables, features extraction and dimension reduction, model, training and evaluation metrics.*
